# A Rare Presentation of HIV-Negative Plasmablastic Lymphoma: A Diagnostic Dilemma

**DOI:** 10.1155/2019/2907317

**Published:** 2019-02-13

**Authors:** Alaina J. Kessler, Bridget K. Marcellino, Scot A. Niglio, Bruce E. Petersen, Adriana K. Malone

**Affiliations:** ^1^Department of Medicine, Icahn School of Medicine at Mount Sinai, 1 Gustave L. Levy Place, New York, NY 10029, USA; ^2^Tisch Cancer Institute, Icahn School of Medicine at Mount Sinai, New York, NY, USA; ^3^Department of Pathology, Icahn School of Medicine at Mount Sinai, New York, NY, USA

## Abstract

Plasmablastic lymphoma (PBL) and plasmablastic plasma cell myeloma (PCM) have many overlapping characteristics. Clinical correlation can help make the distinction between the two entities. Human immunodeficiency virus- (HIV-) negative PBL is a rare disease, making the diagnosis more challenging. While there is no standard of care for PBL, current recommendations include dose-adjusted EPOCH (etoposide, vincristine, doxorubicin, cyclophosphamide, and prednisone), with or without bortezomib. We report an aggressive case of HIV-negative plasmablastic lymphoma and discuss the challenge in establishing a diagnosis. We review the literature regarding this disease and current recommendations for treatment.

## 1. Introduction

Plasmablastic lymphoma (PBL), a variant of diffuse large B-cell lymphoma (DLBCL), and plasmablastic plasma cell myeloma (PCM) have many overlapping features, often relying on clinical factors to make the distinction between the two entities. While the majority of cases of PBL are described in patients with human immunodeficiency virus (HIV), HIV-negative PBL is a rare disease associated with poor outcomes, making the diagnosis even more challenging [1]. The distinction between PBL and PCM is important to guide treatment. Although there is no current standard of care for PBL, the most recent literature recommends dose-adjusted EPOCH (etoposide, vincristine, doxorubicin, cyclophosphamide, and prednisone), with or without bortezomib, as first-line therapy [2]. Stem cell transplant (SCT) should be considered for chemosensitive patients [2]. This case highlights an aggressive presentation of a rare entity, HIV-negative PBL, and the challenges of diagnosis and treatment.

## 2. Case Presentation

An 81-year-old male with a history of heart failure with reduced ejection fraction, coronary artery disease with a history of coronary artery bypass grafting, atrial fibrillation (on warfarin), chronic obstructive lung disease, and diabetes mellitus presented to the emergency department with worsening shortness of breath. Two weeks prior to presentation, he had experienced sharp left-sided abdominal pain, which resolved without intervention. Approximately one week prior to presentation, he reported increased dyspnea and orthopnea, which remained present on admission. Additionally, he reported numerous episodes of spontaneous epistaxis for the past week.

On presentation to the emergency department, he was afebrile but tachycardic, tachypneic, and hypoxic to 83% on room air. No neurologic deficits were noted. Initial laboratory examination showed a white blood cell count of 21,500 cells/mm^3^ (reference range 4,500–11,000 cells/mm^3^) with 5% atypical lymphocytes, 22% band cells, 5% metamyelocytes, 2% myelocytes, a hemoglobin of 12.1 g/dL (reference range 13.9–16.3 g/dL), and a platelet count of 42,000/*μ*L (reference range 150,000–450,000/*μ*L). The INR was 4.4, PT was 40.9 seconds (reference range 12.3–14.0 seconds), and APTT was 46.3 seconds (reference range 25.4–34.9 seconds). Chemistries were notable for a creatinine of 3.4 mg/dL (baseline 1.5 mg/dL), total protein 6.5 g/dL (reference range 6.0–8.3 g/dL), albumin 3.2 g/dL (reference range 3.5–4.9 g/dL), AST 310 U/L (reference range 1–35 U/L), ALT 22 U/L (reference range 1–45 U/L), uric acid > 30 mg/dL (reference range 4.0–9.0 mg/dL), and lactate dehydrogenase (LDH) 12,851 U/L (reference range 100–220 U/L). Calcium was within normal limits. Serum free light chain analysis demonstrated elevated free kappa 13,889 mg/L (reference range 3.3–19.4 mg/L) with a kappa/lambda ratio of 491.5 (reference range 0.26–1.65). M-spike was 0.00 g/dL. Testing for HIV was negative. Epstein–Barr virus (EBV) testing was consistent with prior exposure, with EBV IgM negative and IgG positive. Computerized tomography (CT) of the chest, abdomen, and pelvis without contrast showed extensive mediastinal and hilar lymphadenopathy (measuring up to 2 cm), which had increased in size and extent since imaging six months prior which was obtained as routine follow-up for a left lung nodule; no lytic lesions of the bone were identified. He was initiated on dialysis in the setting of worsening renal failure and subsequently intubated for hypoxemic respiratory failure. Bronchoscopy revealed diffuse alveolar hemorrhage.

**Figure 1:**
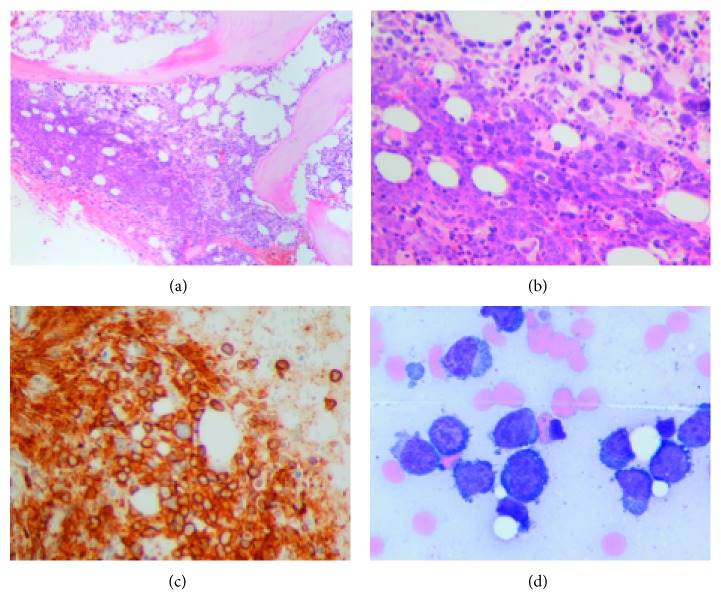
(a, b) Bone marrow biopsy with extensive infiltration by atypical plasmacytoid cells (original magnifications: ×100; ×400). (c) The cells are positive for CD138 by immunohistochemistry, indicative of plasma cell differentiation (original magnification: ×400). (d) Atypical plasmacytoid cells, including forms with plasmablastic morphology, as visualized on bone marrow aspirate smear (original magnification: ×1000).

Peripheral blood flow cytometry showed an aberrant kappa-restricted monotypic plasmacytoid population comprising 7-8% of total (CD138+, CD38 bright+, cytoplasmic kappa+, CD19−, CD56 dim+, CD45+, CD20−, and CD22−). Bone marrow biopsy (Figure 1) showed a hypercellular marrow (60–70%), with an infiltrate of markedly pleomorphic kappa-restricted monotypic plasmacytoid cells, many with immature features, comprising approximately 50% of overall cellularity (CD138+, kappa+, lambda−, CD117 focal+, CD56−, CD20−, and TdT−, by immunohistochemistry). *In situ* hybridization study for EBV-encoded RNA (EBER) was negative; however, sensitivity may have been limited due to decalcification. Flow cytometry showed an immunophenotype similar to that of the peripheral blood specimen.

Cytogenetic analysis demonstrated 100% of cells with a complex karyotype consisting of a jumping 1q translocation between *t*(1;3), *t*(1;11), and *t*(1;12) resulting in gains of 1q; a balanced translocation between the long arm of chromosomes 8 and 14, associated with MYC-IGH fusion; interstitial deletions of chromosomes 4*p* and 4*q*; gains/partial gains of chromosomes 7 and 12, and a derivative chromosome 21 as a result of an unbalanced translocation *t*(13;21) resulting in three copies of 13q. FISH analysis showed 43% of cells with MYC-IGH [*t*(8;14)] fusion.

The differential diagnosis included plasmablastic lymphoma (PBL) and plasmablastic plasma cell myeloma (PCM). The favored diagnosis of PBL was largely based on clinical factors, including the highly aggressive presentation, lymphadenopathy both above and below the diaphragm, and absence of lytic lesions.

Due to advanced age, comorbidities, and impaired renal function, the decision was made to treat with dose-adjusted V-EPOCH (bortezomib, etoposide, dexamethasone, vincristine, cyclophosphamide, and doxorubicin) with the plan for 50% dose reduction of etoposide, doxorubicin, and vincristine and 25% dose reduction of cyclophosphamide on account of the patient being in acute renal failure. The patient received bortezomib (1.3 mg/m^2^ on day 1), doxorubicin (5 mg/m^2^ on days 1 and 2), etoposide (25 mg/m^2^ on days 1 and 2), and vincristine (0.2 mg/m^2^ on days 1 and 2). After two days of chemotherapy, he was noted to have unequal pupillary size. Magnetic resonance imaging (MRI) of the brain revealed watershed temporal lobe infarctions, and further chemotherapy was held. Based on his family's wishes, the patient was transitioned to comfort care measures and transferred to the palliative care unit. He was palliatively extubated and died 12 hours later.

## 3. Discussion

The case presented here exemplifies the difficulty of distinguishing between PBL, a variant of diffuse large B-cell lymphoma (DLBCL), and PCM with extramedullary involvement. Distinguishing between the two can be difficult as the two entities have many overlapping characteristics. It is imperative however to make this diagnostic distinction because the two entities are treated differently.

PBL and PCM have similar morphological and immunophenotypic features but subtle histological differences have been noted by Vega et al. [3]. PBL typically demonstrates a proliferation of plasmablasts and immunoblasts with rare cells showing mature plasmacytic differentiation. In contrast, cells with plasmacytic differentiation are typically more numerous in PCM. The two entities also have similar immunophenotypic profiles with both expressing plasma cell markers, such as CD38, CD138, and MUM1, without classic B-cell markers, such as CD19, CD20, and PAX-5 [3]. Monotypic light chain expression has been demonstrated in both PBL and PCM [3]. Complex karyotypes have also been shown to be associated with both entities [4]. Rearrangements in MYC, an oncogene originally described in Burkitt lymphoma, have been associated with both tumor progression in multiple myeloma as well as plasmablastic lymphoma [1, 4]. Despite these similar features, genomic profiling suggests PBL may be more closely related to DLBCL based on segmental gains such as 16p13.3 frequently seen in both diseases. [5].

Due to the overlapping pathologic features of PBL and PCM, clinical correlation is nearly always required to make the distinction between these two entities. PBL is highly associated with viral reactive lymphadenopathies such as HIV and EBV [3, 6, 7]. EBV positivity is detected more frequently in HIV-positive patients (75%) and posttransplant PBL (67%) compared to PBL in immunocompetent patients (50%) [1]. This patient was HIV-negative, and presence or absence of EBV could not be firmly established. While some criteria for multiple myeloma were present including anemia, renal insufficiency, and elevated free light-chain ratio, the patient lacked the presence of lytic lesions and hypercalcemia that would have favored a diagnosis of multiple myeloma [8]. Ultimately, the diagnosis of PBL was favored over PCM due to the aggressive presentation associated with diffuse lymphadenopathy, elevated LDH, and hyperuricemia.

Although the actual incidence of HIV-negative PBL is unknown, the limited numbers of published cases suggest it is a rare entity. The largest case review from 1997 to 2014 revealed only 164 cases of HIV-negative PBL [2]. HIV-negative PBL is a male-predominant malignancy with a median age of 55 years [2]. The majority (75%) of patients with HIV-negative PBL are immunocompetent although PBL has been associated with lymphoproliferative and autoimmune disorders [9]. PBL has also been identified after solid organ transplantation [9]. A review of 76 patients with HIV-negative PBL showed the majority (89%) of patients presented with extranodal involvement, including disease in the oral cavity (21%), gastrointestinal tract (20%), soft tissue (17%), and bone marrow (15%) [9]. Poor prognostic factors include immunosuppression, Ann Arbor stage IV, EBV negativity, disease refractory to treatment, and C-MYC aberrations [1, 10]. Median overall survival of immunocompetent patients with PBL ranged from 11 to 19 months [1, 10].

While plasmablastic plasma cell myeloma is initially treated with VRd (bortezomib, lenalidomide, and dexamethasone), there is no standard of care for PBL [2, 11]. Given the poor outcomes and lack of response to commonly used chemotherapy such as CHOP (cyclophosphamide, doxorubicin, vincristine, and prednisone) and CHOP-like regimens, the National Comprehensive Cancer Network (NCCN) guidelines state that standard CHOP is not adequate treatment [12]. The NCCN recommends dose-adjusted EPOCH (etoposide, vincristine, doxorubicin, cyclophosphamide, and prednisone), CODOX-M/IVAC (cyclophosphamide, vincristine, doxorubicin, methotrexate alternating with ifosfamide, etoposide, and cytarabine), or Hyper-CVAD (hyperfractionated cyclophosphamide, vincristine, doxorubicin, and dexamethasone alternating with methotrexate and cytarabine).

Recent studies have focused on new therapeutic approaches, specifically the use of bortezomib, a proteasome inhibitor approved for the treatment of multiple myeloma and mantle cell lymphoma. There are limited case reports using bortezomib in patients with HIV-negative PBL. A recent review of the literature through March 2017 by Guerrero-Garcia et al. [13] identified 21 patients, ten of which (48%) were HIV-negative PBL. All had extranodal involvement. Of these ten patients, three (30%) patients achieved complete response and seven (70%) patients had partial response. Also of note in the frontline setting, the three patients (60%) who achieved complete response had received bortezomib as part of CYBORD (cyclophosphamide, bortezomib, and dexamethasone) or in combination with EPOCH. A recently published case report of PBL involving the right parotid gland in a HIV-negative patient showed continuous complete remission at 12 months using bortezomib plus lenalidomide upon relapse after CHOP [14]. Another case report of HIV-negative PBL utilizing a lenalidomide-based regimen with complete remission at 24 months suggests its usage as an alternative therapy for patients unable to tolerate intensive chemotherapy [8].

Stem cell transplant (SCT) should be considered for chemosensitive patients. A small case series of nine patients with HIV-negative PBL in which all were treated with CHOP or hyper-CVAD showed that seven patients achieved complete response and one patient achieved a partial response. [15] Three of the seven patients who achieved complete response and one patient who achieved partial response underwent consolidation with high-dose chemotherapy and autologous hematopoietic SCT. Of these four patients, two had no evidence of disease at 24 months. Although a small case series, these findings suggest that SCT can obtain durable and prolonged responses. The suggested treatment by Castillo et al. [2] is six cycles of infusional dose-adjusted EPOCH, with or without bortezomib, accompanied by intrathecal prophylaxis with each cycle of EPOCH as first-line therapy. For appropriate candidates, those authors also recommend consolidative high-dose chemotherapy followed by autologous SCT for patients in first remission. In patients who are chemorefractory in whom autologous SCT is not feasible, there is a single case report of an allogeneic SCT with cord blood transplantation achieving durable remission [16]. Recent data show SLAMF2 (CD319/CS1), targetable by elotuzumab, is expressed in PBL and has been proposed as a potential therapeutic target [17].

In conclusion, this case highlights an aggressive presentation of a rare entity, HIV-negative PBL, and discusses the clinical dilemma arriving at the diagnosis. As with this case, clinical correlation is frequently required to make the diagnosis. The distinction between these two entities is important for guiding treatment.
